# Supplementary material for: Genome-wide identification, characterization and gene expression of BES1 transcription factor family in grapevine (Vitis vinifera L.)
Source: Sci Rep. 2023 Jan 5;13:240. doi: 10.1038/s41598-022-24407-y (PMC9816167; doi:10.1038/s41598-022-24407-y)
Supplement: Supplementary file 3 — Supplementary Information. [file 41598_2022_24407_MOESM3_ESM.zip › Vvi_Atr/Vitis_vinifera.PN40024.v4.dna_sm.toplevel.fa.vs.Amborella_trichopoda.AMTR1.0.dna_sm.toplevel.fa.html/Atr-AmTr_v1.0_scaffold00162.html]

|  |  |  |  |  |  |  |  |  |  |  |  |  |  |
| --- | --- | --- | --- | --- | --- | --- | --- | --- | --- | --- | --- | --- | --- |
| Duplication depth | Reference chromosome | Collinear blocks | | | | | | | | | | | |
| 0 | Atr-ERN09331 |  |  |  |  |  |  |  |  |  |  |  |  |
| 0 | Atr-ERN09332 |  |  |  |  |  |  |  |  |  |  |  |  |
| 0 | Atr-ERN09333 |  |  |  |  |  |  |  |  |  |  |  |  |
| 0 | Atr-ERN09334 |  |  |  |  |  |  |  |  |  |  |  |  |
| 0 | Atr-ERN09335 |  |  |  |  |  |  |  |  |  |  |  |  |
| 0 | Atr-ERN09336 |  |  |  |  |  |  |  |  |  |  |  |  |
| 0 | Atr-ERN09337 |  |  |  |  |  |  |  |  |  |  |  |  |
| 0 | Atr-ERN09338 |  |  |  |  |  |  |  |  |  |  |  |  |
| 1 | Atr-ERN09339 |  | Vvi-Vitvi10g00660\_t001 |  |  |  |  |  |  |  |  |  |  |
| 1 | Atr-ERN09340 |  | \| \| |  |  |  |  |  |  |  |  |  |  |
| 1 | Atr-ERN09341 |  | \| \| |  |  |  |  |  |  |  |  |  |  |
| 1 | Atr-ERN09342 |  | \| \| |  |  |  |  |  |  |  |  |  |  |
| 1 | Atr-ERN09343 |  | \| \| |  |  |  |  |  |  |  |  |  |  |
| 1 | Atr-ERN09344 |  | \| \| |  |  |  |  |  |  |  |  |  |  |
| 1 | Atr-ERN09345 |  | \| \| |  |  |  |  |  |  |  |  |  |  |
| 1 | Atr-ERN09346 |  | \| \| |  |  |  |  |  |  |  |  |  |  |
| 1 | Atr-ERN09347 |  | \| \| |  |  |  |  |  |  |  |  |  |  |
| 1 | Atr-ERN09348 |  | \| \| |  |  |  |  |  |  |  |  |  |  |
| 1 | Atr-ERN09349 |  | \| \| |  |  |  |  |  |  |  |  |  |  |
| 1 | Atr-ERN09350 |  | \| \| |  |  |  |  |  |  |  |  |  |  |
| 1 | Atr-ERN09351 |  | \| \| |  |  |  |  |  |  |  |  |  |  |
| 1 | Atr-ERN09352 |  | \| \| |  |  |  |  |  |  |  |  |  |  |
| 1 | Atr-ERN09353 |  | Vvi-Vitvi10g00657\_t001 |  |  |  |  |  |  |  |  |  |  |
| 1 | Atr-ERN09354 |  | \| \| |  |  |  |  |  |  |  |  |  |  |
| 1 | Atr-ERN09355 |  | \| \| |  |  |  |  |  |  |  |  |  |  |
| 1 | Atr-ERN09356 |  | Vvi-Vitvi10g00641\_t001 |  |  |  |  |  |  |  |  |  |  |
| 1 | Atr-ERN09357 |  | \| \| |  |  |  |  |  |  |  |  |  |  |
| 1 | Atr-ERN09358 |  | \| \| |  |  |  |  |  |  |  |  |  |  |
| 1 | Atr-ERN09359 |  | \| \| |  |  |  |  |  |  |  |  |  |  |
| 1 | Atr-ERN09360 |  | \| \| |  |  |  |  |  |  |  |  |  |  |
| 1 | Atr-ERN09361 |  | \| \| |  |  |  |  |  |  |  |  |  |  |
| 1 | Atr-ERN09362 |  | \| \| |  |  |  |  |  |  |  |  |  |  |
| 1 | Atr-ERN09363 |  | \| \| |  |  |  |  |  |  |  |  |  |  |
| 1 | Atr-ERN09364 |  | Vvi-Vitvi10g00640\_t001 |  |  |  |  |  |  |  |  |  |  |
| 1 | Atr-ERN09365 |  | \| \| |  |  |  |  |  |  |  |  |  |  |
| 1 | Atr-ERN09366 |  | Vvi-Vitvi10g00639\_t001 |  |  |  |  |  |  |  |  |  |  |
| 1 | Atr-ERN09367 |  | Vvi-Vitvi10g00638\_t001 |  |  |  |  |  |  |  |  |  |  |
| 0 | Atr-ERN09368 |  |  |  |  |  |  |  |  |  |  |  |  |
| 0 | Atr-ERN09369 |  |  |  |  |  |  |  |  |  |  |  |  |
